# The Cystic Fibrosis Upper and Lower Airway Metagenome

**DOI:** 10.1128/spectrum.03633-22

**Published:** 2023-03-09

**Authors:** Katarzyna Pienkowska, Marie-Madlen Pust, Margaux Gessner, Svenja Gaedcke, Ajith Thavarasa, Ilona Rosenboom, Patricia Morán Losada, Rebecca Minso, Christin Arnold, Silke Hedtfeld, Marie Dorda, Lutz Wiehlmann, Jochen G. Mainz, Jens Klockgether, Burkhard Tümmler

**Affiliations:** a Department for Pediatric Pneumology, Allergology and Neonatology, Hannover Medical School, Hannover, Germany; b Biomedical Research in Endstage and Obstructive Lung Disease, German Center for Lung Research, Hannover, Germany; c Cystic Fibrosis Center for Children and Adults, Jena University Hospital, Jena, Germany; d Research Core Unit Genomics, Hannover Medical School, Hannover, Germany; e Klinik für Kinder- und Jugendmedizin, Medizinische Hochschule Brandenburg, Brandenburg, Germany; Emory University School of Medicine

**Keywords:** airways, cystic fibrosis, metagenomics

## Abstract

The microbial metagenome in cystic fibrosis (CF) airways was investigated by whole-genome shotgun sequencing of total DNA isolated from nasal lavage samples, oropharyngeal swabs, and induced sputum samples collected from 65 individuals with CF aged 7 to 50 years. Each patient harbored a personalized microbial metagenome unique in microbial load and composition, the exception being monocultures of the most common CF pathogens Staphylococcus aureus and Pseudomonas aeruginosa from patients with advanced lung disease. The sampling of the upper airways by nasal lavage uncovered the fungus Malassezia restricta and the bacterium Staphylococcus epidermidis as prominent species. Healthy and CF donors harbored qualitatively and quantitatively different spectra of commensal bacteria in their sputa, even in the absence of any typical CF pathogen. If P. aeruginosa, S. aureus, or Stenotrophomonas maltophilia belonged to the trio of the most abundant species in the CF sputum metagenome, common inhabitants of the respiratory tract of healthy subjects, i.e., Eubacterium sulci, Fusobacterium periodonticum, and Neisseria subflava, were present only in low numbers or not detectable. Random forest analysis identified the numerical ecological parameters of the bacterial community, such as Shannon and Simpson diversity, as the key parameters that globally distinguish sputum samples from CF and healthy donors.

**IMPORTANCE** Cystic fibrosis (CF) is the most common life-limiting monogenetic disease in European populations and is caused by mutations in the *CFTR* gene. Chronic airway infections with opportunistic pathogens are the major morbidity that determines prognosis and quality of life in most people with CF. We examined the composition of the microbial communities of the oral cavity and upper and lower airways in CF patients across all age groups. From early on, the spectrum of commensals is different in health and CF. Later on, when the common CF pathogens take up residence in the lungs, we observed differential modes of depletion of the commensal microbiota in the presence of S. aureus, P. aeruginosa, S. maltophilia, or combinations thereof. It remains to be seen whether the implementation of lifelong CFTR (cystic fibrosis transmembrane conductance regulator) modulation will change the temporal evolution of the CF airway metagenome.

## INTRODUCTION

Cystic fibrosis (CF) is a severe monogenic trait with autosomal recessive inheritance that is caused by mutations in the *CFTR* gene (1). The basic defect of impaired chloride and bicarbonate secretion predisposes the individual to chronic airway infections (2). The CF lungs typically harbor polymicrobial communities with dozens of bacterial species, viruses, and fungi, with Staphylococcus aureus and Pseudomonas aeruginosa being the most prevalent hallmark pathogens (3–10).

The composition of the bacterial communities in CF airways has so far mainly been studied by 16S rRNA gene amplicon sequencing (5–8, 10–32). Alternatively, whole-genome shotgun (WGS) metagenome sequencing can be performed (33), where host and microbial DNA is extracted and sequenced from biological specimens with no or low amplification. Microbial communities are then determined and defined via reference-based alignment and analysis of the sequenced nonhost DNA. WGS metagenomics is “taxonomically agnostic” and can identify fungi, archaea, eubacteria, and DNA viruses, if proper reference genome sequences are available for the alignment.

The focus of past microbial metagenome studies investigating the lower CF airway has been on small cohorts of CF adolescents and adults who naturally expectorate relatively large volumes of respiratory secretions (34–48). We have optimized our protocols so that deep microbial metagenome sequencing has become feasible with samples retrieved from all age groups (3, 49, 50). This achievement laid the foundation to conduct a representative survey of the airway microbiome of the current CF patient population across all age groups.

In this study, we compared the microbial metagenome of the oral cavity, the sinonasal compartment, and lower airway. The study revealed that the CF host and the corresponding disease status were the major determinants of the quantitative taxonomic profile of the CF airway metagenome. Each CF patient over the age of 6 years harbored a personalized microbial metagenome, which was unique in microbial load and composition. Notable exceptions to this observation were monocultures of the most common CF pathogens, S. aureus and P. aeruginosa, in patients with advanced lung disease. The host-driven individual signature was shared by the oral cavity and lower airway.

## RESULTS

### Patients.

**FIG 1:**
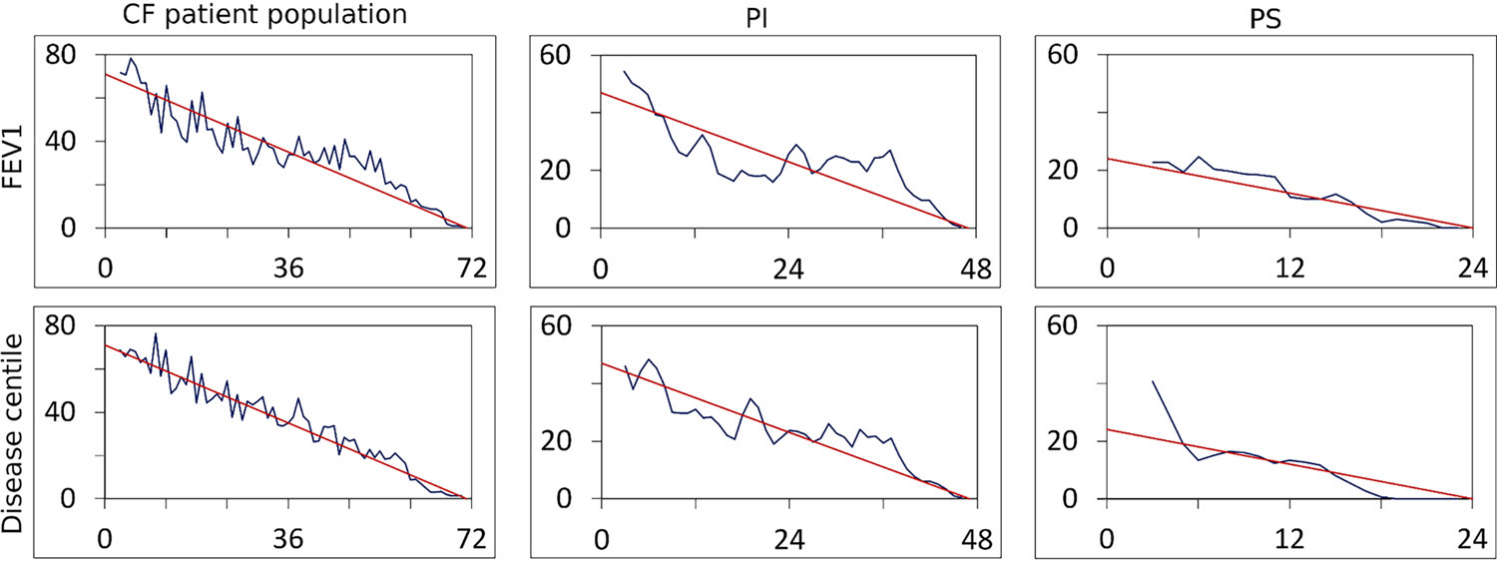
The CF patient cohort lung function and disease centile distribution matches the centile distribution of the CF registry. Frequency of centile number differences normalized to cohort size (ordinate) is plotted against the rank number difference for the selected patient cohort (in blue) compared to the expected theoretical linear decrease (in red). Graphs represent the FEV1 centile (top) and the disease centile (bottom) for (left to right) the whole cohort and the PI and PS patient subgroups. Patients’ FEV1 and disease centiles were determined by mapping the individuals’ data onto the European CF Registry centiles (41) as a reference. The study cohort’s centile distribution was not significantly different from the expected linear decline.

Nasal lavage samples, oropharyngeal swabs, and induced sputa (Fig. S1) were collected on one to seven occasions within a period of up to 25 months from 45 exocrine pancreatic-insufficient (PI) and 20 exocrine pancreatic-sufficient (PS) individuals with CF (Table 1; also, see Table S1 in the supplemental material). PI patients carry two severe disease-causing *CFTR* mutations in the *CFTR* gene that confer no or only minimal CFTR (cystic fibrosis transmembrane conductance regulator) function, whereas PS patients harbor at least one *CFTR* allele that confers residual CFTR activity in the range of 10 to 20% of the wild-type level and is typically associated with a milder course of CF disease (1, 51). The distribution of lung function and disease centiles of the study cohort matched with the age- and gender-corrected centile distribution of the patient registry of the European Cystic Fibrosis Society (52) (Fig. 1), implying that our study cohort is representative of the disease severity of the current CF patient population. The patient population consisted of 34 children and adolescents (6 to 17 years) and of 31 adults (18 to 50 years) (Data Sets S1 and S2). Induced sputum samples and oropharyngeal swabs were also collected from 157 schoolchildren, adolescents, and adults who, like the CF patient cohort, were living in Central Lower Saxony in Germany (Table S2).

**TABLE 1:** CF patient cohort characteristics

Characteristic	Value for group		
	All (*n* = 65)	PI (*n* = 45)	PS (*n* = 20)
No. (%) of females	31 (48)	21 (47)	10 (50)
Mean age (SD) (yrs)	21.4 (10.3)	22.1 (10.9)	19.6 (8.6)
No. (%) in age group			
7–17 yrs	34 (52)	22 (49)	12 (60)
18–27 yrs	13 (20)	8 (18)	5 (25)
28–50 yrs	18 (28)	15 (33)	3 (15)
FEV1 centile^*a*^	44 (27–75; 3–99)	32 (15–68; 3–99)	50 (39–76; 13–99)
BMI centile^*a*^	48 (26–70; 6–99)	40 (21–73; 7–99)	50 (30–61; 6–91)
Disease centile^*a*^	52 (28–71; 9–94)	42 (24–72; 9–94)	60 (34–71; 15–84)

a Median (inner quartile; range).

### Microbial metagenome of nasal lavage samples, oropharyngeal swabs, and induced sputum of the CF cohort.

**FIG 2:**
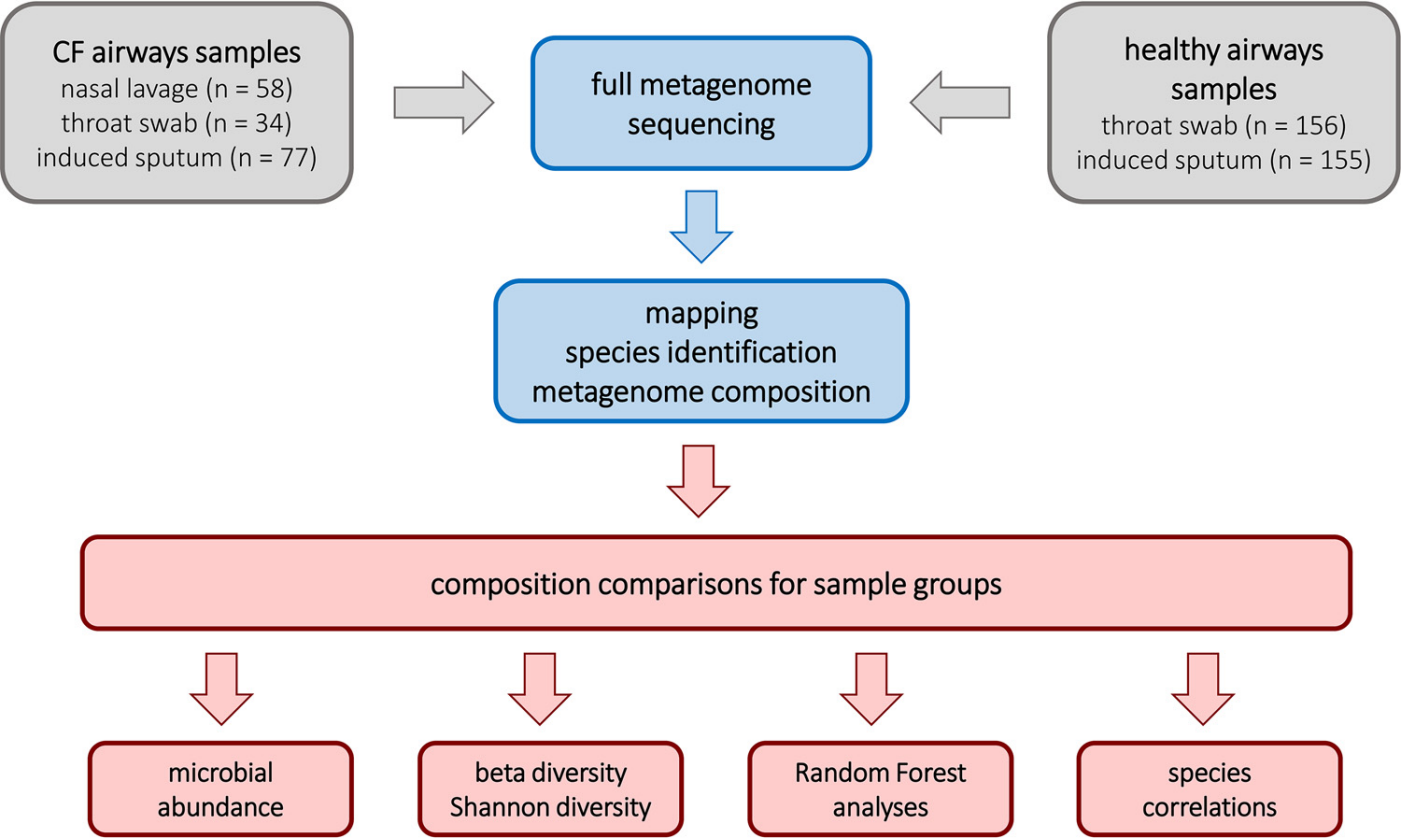
Flow chart of the processing and evaluation of airway samples from CF and healthy non-CF donors.

Genomic DNA isolated from nasal lavage samples, oropharyngeal swabs, and induced sputum was subjected to high-throughput shotgun metagenome sequencing. After processing, trimming, and quality filtering, the primary reads were mapped onto the human reference genome and a microbial pangenome of completely sequenced genomes (see Materials and Methods) (Fig. 2). The normalized read numbers were deposited in the local database (Data Sets S1 and S2) representing the number of reads per million base pairs of reference genome per million reads (RPMM) of species and higher taxonomic ranks. Selected samples were reinvestigated by quantitative PCR (qPCR).

**FIG 3:**
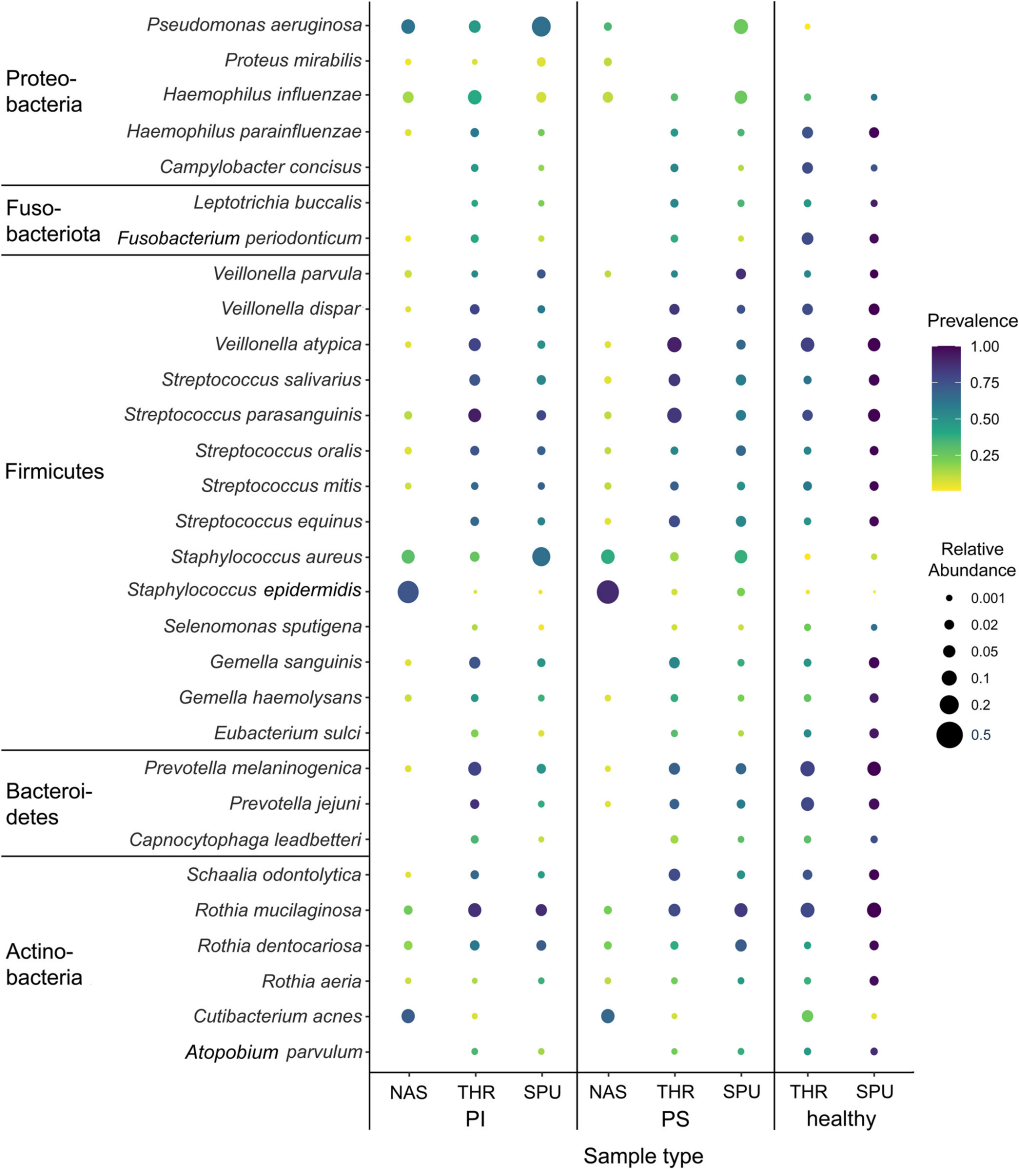
Bubble graph presentations of the abundance and prevalence of bacteria in airway metagenome data sets in health and cystic fibrosis. Induced sputum (SPU), oropharyngeal swabs (THR), and nasal lavage samples (NAS) were collected from PI CF and PS CF patients and healthy non-CF controls. For each habitat and condition, the area of the circle indicates the mean relative abundance and the color intensity indicates the prevalence of the species within the group. The scales on the right visualize the calibrated color intensity gradient of prevalence and the calibrated range of the abundance. The selected bacterial species account for 90% of the total number of reads among archaebacteria and eubacteria.

**FIG 4:**
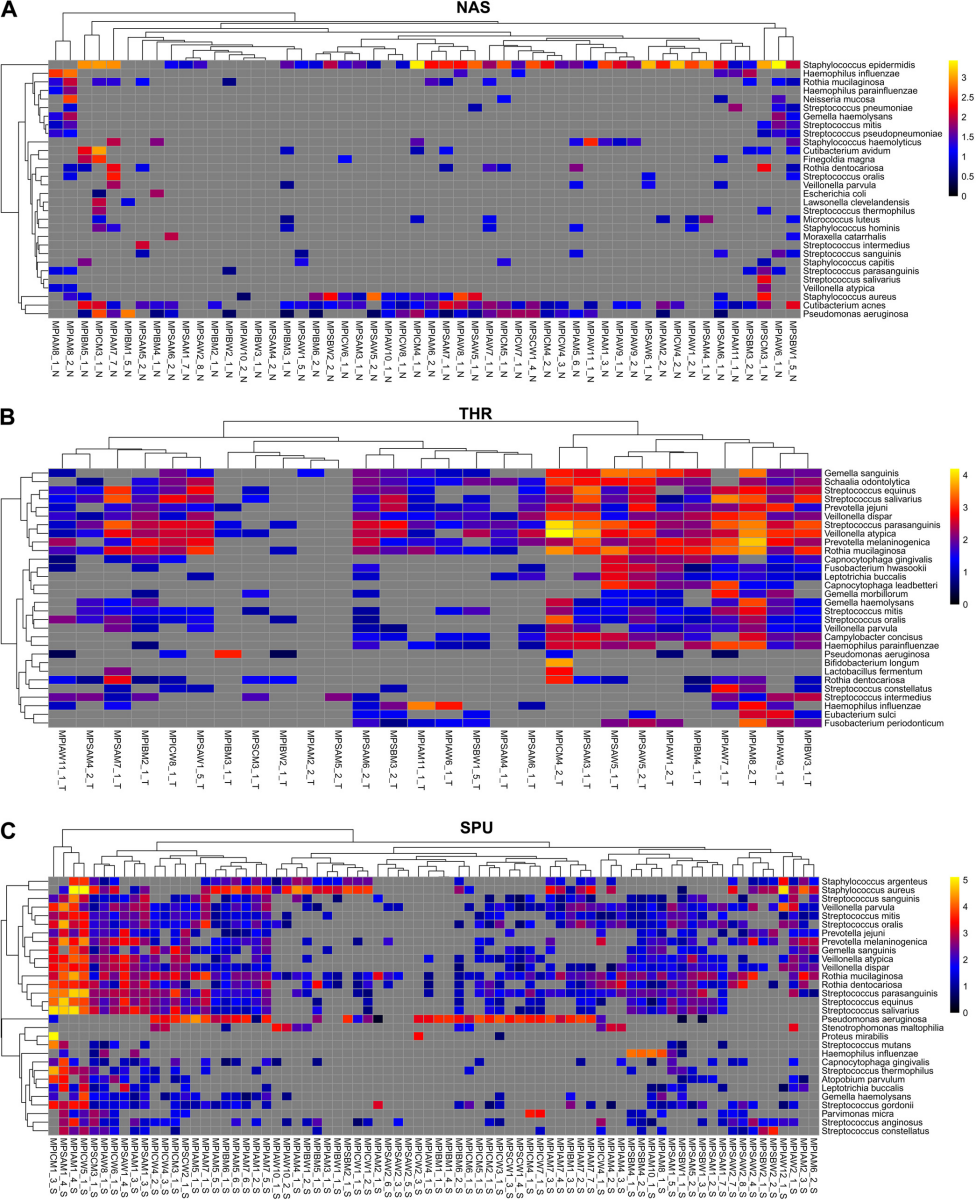
Heat maps of the top 30 bacterial species in nasal lavage samples (NAS) (A), oropharyngeal swabs (THR) (B), and sputa (SPU) (C) collected from PI and PS patients. The color indicates the absolute abundance of a bacterial taxon in the sample’s metagenome. Sample identifiers include characters indicating (from left to right) origin (M, CF clinic in Hannover), pancreatic status (PI or PS), age group (A, 7 to 17 years; B, 18 to 27 years; C, 28 to 50 years), gender (M, male; W, female), patient number, sample number, and sample type (N, nasal lavage sample; S, sputum; T, throat swab). Absolute abundance is shown by the color code at logarithmic scale: log_10_(RPMM + 1). A gray square indicates that the taxon was not detected in the sample.

The microbial metagenome was dominated by bacteria, which on average made up more than 99% of microbial reads (Data Sets S1 and S2). The sampling of the upper airways by nasal lavage uncovered two prominent bacterial species, i.e., predominantly Staphylococcus epidermidis and to a lesser extent *Cutibacterium* (*Propionibacterium*) *acnes.* In two of three patient samples, they accounted for 10% to more than 90% of the microbial community (Fig. 3 and 4A). S. epidermidis and C. acnes are common members of the skin flora (53), but are not typical inhabitants of healthy nasal conchae. In CF, however, the inherited basic defect predisposes to rhinosinusitis, inflamed nasal passages, or a stuffy nose (1, 54, 55) which apparently triggers the upper airway colonization with these skin commensals. The representatives of a healthy nasal microbiome (56, 57), including Streptococcus, *Prevotella*, *Veillonella*, *Rothia*, and *Neisseria* species, were the major constituents of diversity, each having a share of a few percent. The typical CF pathogens S. aureus and P. aeruginosa were present in the majority of nasal lavage samples in similar quantities as *C. acnes.* In most samples, they were only minor members of the community. Only in one-fifth of samples was the relative abundance higher than 10%.

Oropharyngeal swabs recovered a similar pattern of commensal *Firmicutes*, *Fusobacteria*, *Bacteroidetes*, and *Actinobacteria* species from healthy controls and PI and PS patients (Fig. 3 and 4B). The typical CF pathogens S. aureus, P. aeruginosa, and Haemophilus influenzae were present in relatively low numbers, albeit more abundant and more prevalent in PI patients’ than PS patients’ samples.

This difference between PI and PS CF was even more pronounced in induced sputum, which has been demonstrated to be an appropriate proxy for the respiratory secretions that line the conducting lower airway (31, 58). The metagenome of the PI CF cohort was dominated by S. aureus and P. aeruginosa, consistent with our common knowledge of CF microbiology derived from culture-dependent diagnostics. The PS CF lower-airway bacteriome was between those of PI CF patients and healthy controls (Fig. 3 and 4C).

Only 0.1% of the DNA reads per sample were on average assigned to fungi and molds. For technical reasons, the contribution of the mycobiome to the CF airway metagenome was most likely underestimated, because, first, our DNA extraction protocol was not adapted to the lysis of rigid fungal cell walls and, second, a representative reference mycobiome could not be compiled from the databases due to the continuing paucity of completely sequenced fungal genomes. Applying the tool EukDetect (59), we identified 10 fungal species altogether in 36 sputa from 3 of 20 PS patients and from 14 of 45 PI patients (Table 2). Consistent with the CF literature, Aspergillus fumigatus and Exophilia dermatitis were most often detected. More than one fungus was detected in samples from six patients. Numerous nasal lavage samples contained the sinus-associated fungi Malassezia restricta and Malassezia globosa (Table 2) (60).

**TABLE 2:** Mycobiome of CF patients’ samples ascertained by EukDetect

Sample and fungal species	No. of:	
	CF donors	Samples
Nasal lavage		
*Bradysia coprophila*	1	1
*Cyberlindnera jadinii*	1	1
*Malassezia globosa*	2	2
*Malassezia restricta*	22	22
Induced sputum		
Aspergillus fumigatus	5	6
Candida albicans	4	6
Candida dubliniensis	1	1
Candida glabrata	2	3
Clavispora lusitaniae	2	2
Cyberlindnera jadinii	1	1
Exophiala dermatitidis	5	7
Saccharomyces cerevisiae	3	3
Scedosporium apiospermum	1	3
Scedosporium boydii	2	2

### Beta diversity.

**FIG 5:**
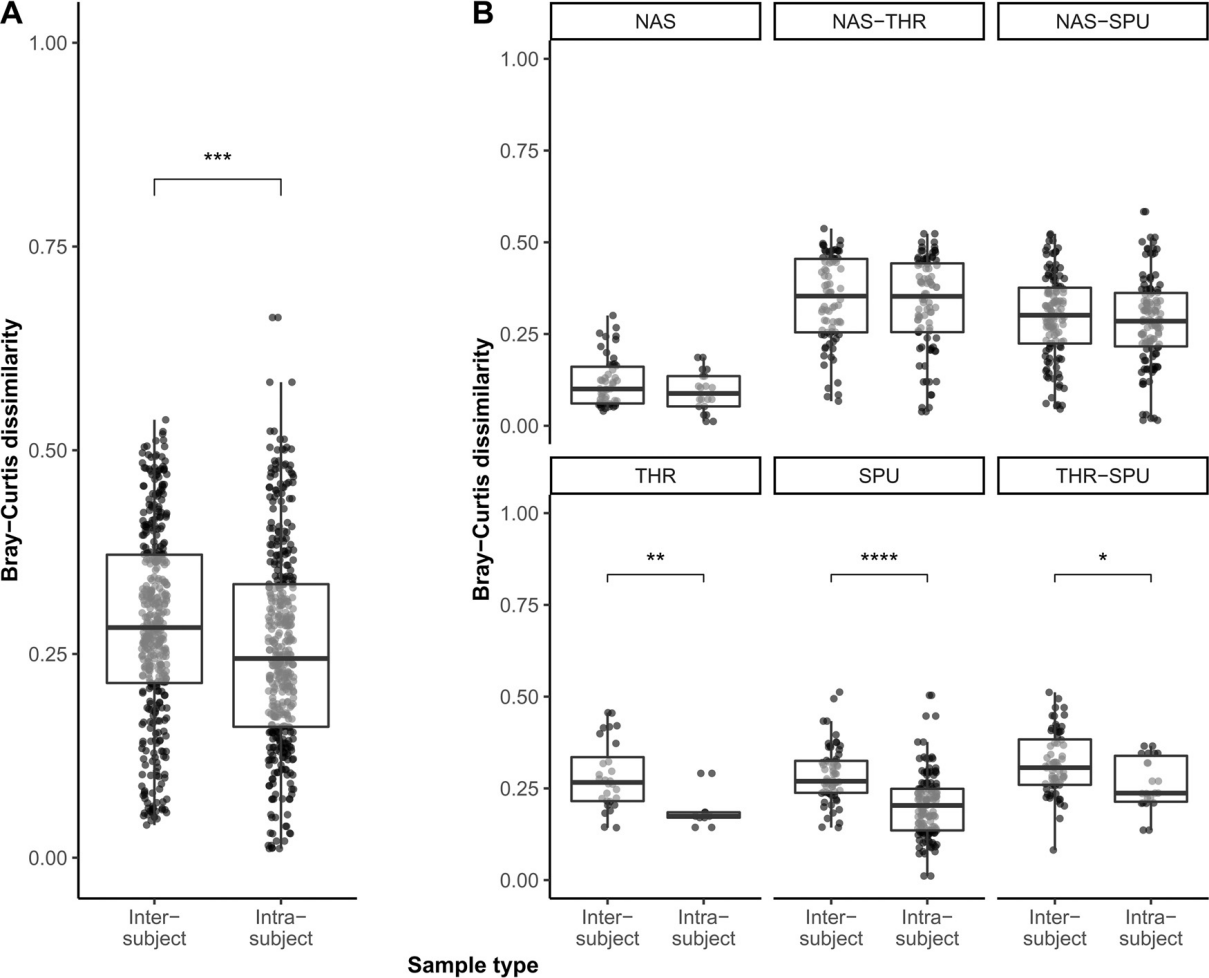
Multipanel box plot comparisons of intra- and intersubject metagenome dissimilarities based on 400 randomly selected pairwise Bray-Curtis distance combinations across ecological niches in the CF airway habitat. (A) In general, with respect to the metagenome composition, the longitudinal samples (intrasubject) were more similar to each other than cross-sectional intersubject samples (Wilcoxon test, *P* < 0.001, *r* = 0.13, CI = 0.06 to 0.2). (B) While the metagenome compositions of interpatient and intrapatient nasal lavage samples (top) were found to be similar (NAS, Wilcoxon test, *P* > 0.05), interpatient sputum samples (SPU; Wilcoxon test, *P* < 0.0001, *r* = 0.44, CI = 0.33 to 0.55) and throat swabs (THR; Wilcoxon test, *P* < 0.01, *r* = 0.47, CI = 0.15 to 0.7) were significantly more dissimilar than longitudinal samples from the same patient (bottom). This personalized signature was also detected when assessing the dissimilarity between inter- and intrapatient metagenomes of sputum samples and throat swabs (Wilcoxon test, *P* < 0.05, *r* = 0.26, CI = 0.06 to 0.43).

We performed nonmetric multidimensional scaling to assess the effect of sample type (nasal lavage samples, oropharyngeal swabs, and induced sputa) and clinical variables on the microbial respiratory tract community structure. Therefore, we fitted metadata onto the ordination axes, including age group, gender, pancreatic state, type and donor of sample, lung function (FEV1 [forced expiratory volume in the first second] percentile), disease and BMI percentile. Neither gender nor pancreatic state had a significant effect on the microbial community structures (Table 3). Conversely, lung function was a discriminatory parameter for the ordination (Table 3). Moreover, the individual CF host shaped the microbial community structure. Samples that had been repetitively collected from the same patient over a period of up to 2 years could be discerned from other donors’ samples and clustered together (Table 3; Fig. 5A). In other words, longitudinal intrapatient were more similar than cross-sectional interpatient samples, even across ecological niches in the CF airway habitat (Fig. 5A). This personalized metagenome signature was detected in oropharyngeal swabs and induced sputa (Fig. 5B), confirming previous reports that the CF airway should be considered an open habitat with extensive exchange between upper and lower airways (55). However, the personalized signature in terms of Bray-Curtis dissimilarity indices was not observed in metagenome samples of the nasal conchae (Fig. 5B).

**TABLE 3:** Nonmetric multidimensional scaling^*a*^ based on Bray-Curtis dissimilarity indices^*b*^

Parameter	Goodness of fit	
	*r* ^2^	*P* ^*c*^
Sample type (nasal lavage, oropharyngeal swab, induced sputum)	0.14	<0.01**
Pancreatic state	0.03	>0.05
Age group (7–17, 18–27, 28–50 yrs)	0.05	>0.05
Gender	0.01	>0.05
Serial samples from the same donor	0.75	<0.01**
FEV1 centile	0.22	<0.01**
Disease centile	0.19	<0.01**
BMI centile	0.11	<0.05*

a R, metaMDS, *k* = 3, no autotransform.

b A permutation test (R envfit, number of permutations = 999) was used for plotting metadata variables onto the ordination and obtaining their significance with Benjamini-Hochberg adjustment of *P* values. The stress value of the nonmetric multidimensional scaling ordination (<0.2) indicated a good representation in reduced dimensions.

c *, *P* < 0.05; **, *P* < 0.01.

### Microbial load and disease severity (Fig. 6).

**FIG 6:**
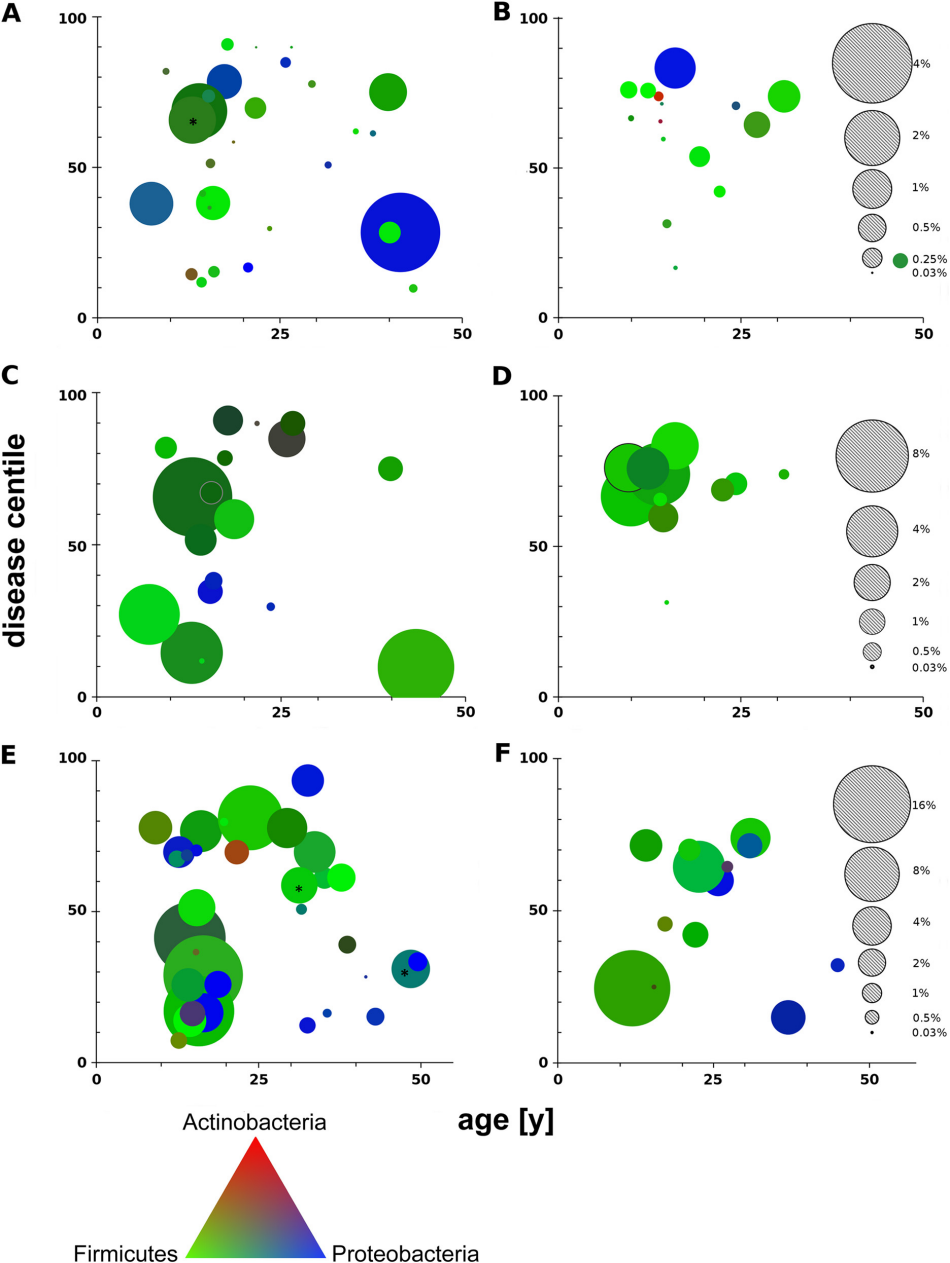
Microbial load (bacterial reads/total reads) of patients’ samples, represented by dot size, is assigned to the patient’s age (*x* axis) and disease centile (*y* axis). The plots visualize the microbial load of nasal lavage samples (A and B), oropharyngeal swabs (C and D) and induced sputa (E and F) collected from PI (left) and PS (right) CF patients. The gray dots indicate the scale of microbial load for each habitat. The RGB color of each dot corresponds to a linear combination of the three dominant phyla: *Actinobacteria* (red), *Firmicutes* (green), and *Proteobacteria* (blue). The triangle at the bottom provides the color reference. Note the different scales for nasal lavage samples, oropharyngeal swabs, and sputa. The dots of the two PI CF sputa with the highest microbial loads, 36% and 40% (asterisks), were reduced by 10-fold (E).

Next, we allocated the individual bacterial metagenome data sets of nasal lavage samples (Fig. 6A and B), oropharyngeal swabs (Fig. 6C and D), and induced sputum (Fig. 6E and F) to the patient’s age and disease centile differentiated by PI (Fig. 6A, C, and E) and PS (Fig. 6B, D, and F) status. Size and color in Fig. 6 visualize the bacterial load and the relative contribution of the three dominant phyla, *Actinobacteria*, *Firmicutes*, and *Proteobacteria*. Each metagenome was unique, as indicated by singular combinations of size and color, the exception being the monocultures of S. aureus and P. aeruginosa (Data Sets S1 and S2). *Firmicutes* were the dominant bacteria in most CF metagenomes of nasal lavage samples, oropharyngeal swabs, and induced sputum. Since shotgun sequencing of all extracted DNA had been performed without depleting the intracellular human DNA, the load of bacterial cells per human cell could be estimated (3). The human genome is approximately about 1,000-fold larger than the average bacterial genome; hence, a contribution of 1% of bacterial reads of total corresponds to about 10 bacterial cells per human cell in a sample (3). Bacterial load in the samples varied by more than 3 orders of magnitude, between 0.1 and 400 bacterial cells per human cell, and increased in the order nasal lavage samples < oropharyngeal swabs < sputum. In contrast to our expectation but consistent with previous reports (13, 61), no association between microbial load and disease severity was observed.

### The lower-airway bacterial community network.

**FIG 7:**
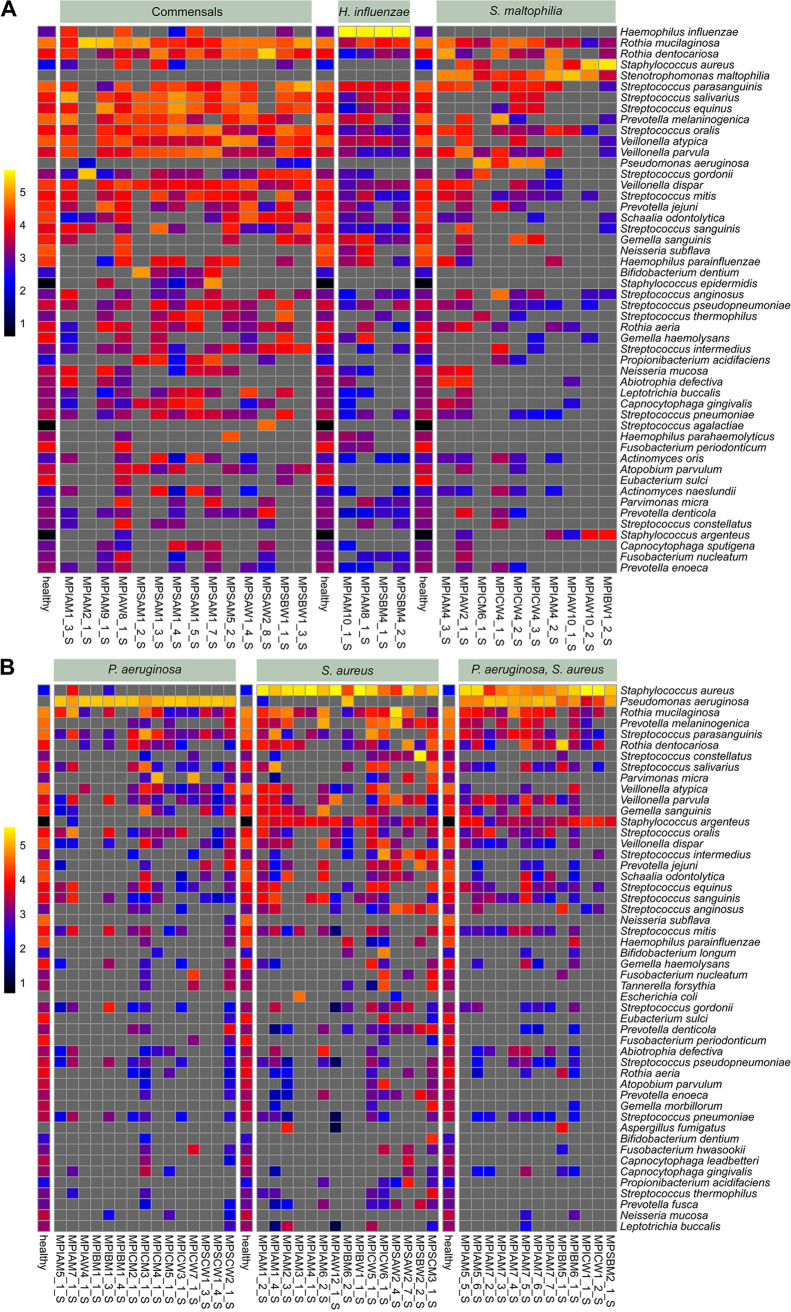
Absolute abundance of the top 50 bacterial species in CF sputa differentiated by the presence of dominant H. influenzae, S. aureus, P. aeruginosa, or S. maltophilia as one of the three most abundant taxa. For each subgroup, the top 50 taxa were compared with the benchmark of a cumulative metagenome of 157 induced sputa from healthy subjects aged 10 to 59 years living in the same geographic region as the CF cohort. Panels show (from left to right) the absence of any dominant CF pathogen followed by the indicated dominant pathogens. Absolute abundance is shown by the color code at logarithmic scale: log_10_(RPMM + 1). A gray square indicates that the taxon was not detected in the sample.

The composition of the patient’s bacterial metagenome, which is visualized in Fig. 6 by its individual color, was resolved in Fig. 4 at the level of the 30 dominant bacterial species. The spectrum of induced sputa as a surrogate for the lower conducting airway ranged from diverse communities of commensals to monocultures of the CF lead pathogen P. aeruginosa. To differentiate the diversity of the lower CF airway bacterial community at higher resolution, subsamples with or without H. influenzae, S. aureus, P. aeruginosa, and Stenotrophomonas maltophilia were compared with the benchmark of a cumulative metagenome of 157 induced sputa from healthy subjects aged 10 to 59 years living in the same geographic region as the CF cohort (Table 4; Fig. 7).

**TABLE 4:** Depletion of common members of the healthy airway metagenome in the CF sputum metagenome in the presence of CF pathogens as one of the three most abundant species

Species^*a*^	Depletion in presence of dominant CF pathogen(s)^*b*^				
	SA	PA	SM	PA and SM	PA and SA
Eubacterium sulci	↓	↓	↓	↓	↓
Fusobacterium periodonticum	↓	↓	↓	↓	↓
Neisseria subflava	↓	↓	↓	↓	↓
Rothia aeria	↓	↓		↓	
Streptococcus pneumoniae	↓		↓	↓	
Streptococcus pseudopneumoniae	↓		↓	↓	
Atopobium parvulum		↓		↓	↓
*Gemella* spp.		↓	↓	↓	↓
*Prevotella* spp.				↓	↓
*Veillonella* spp.				↓	↓
Streptococcus sanguinis					↓
Streptococcus cristatus					↓

a Only species with consistent patterns in all subgroup samples are included.

b SA, S. aureus; PA, P. aeruginosa; SM, S. maltophilia.

Figure 7 displays the absolute abundance of the 50 most common bacterial species in the respective CF subgroup at a logarithmic scale. As expected for a progressive lung disease, all sputa devoid of any typical CF pathogen were collected from patients in the youngest age group. Typical members of the oral microbiome were detected, but the individual patterns of commensals differed from that of the average healthy control (Fig. 7A). The other extreme of no or very few commensal bacteria was seen in a few sputum metagenomes which were dominated by either P. aeruginosa and/or S. aureus or Stenotrophomonas maltophilia. If any of these three pathogens belonged to the trio of the most abundant species in the CF sputum metagenome, common inhabitants of the upper respiratory tract present in all samples of healthy subjects—i.e., Eubacterium sulci, Fusobacterium periodonticum, and Neisseria subflava—were present only in low numbers or not detectable in the CF sputa. Likewise, the presence of S. aureus or P. aeruginosa as the dominant taxon was associated with the suppression of Rothia aeria and Haemophilus parainfluenzae.

Dominance of S. aureus was moreover associated with the reduction of the Streptococcus mitis group, including Streptococcus pneumoniae and Streptococcus pseudopneumoniae, and dominance of P. aeruginosa accompanied suppression of the genera *Gemella*, *Prevotella*, and *Veillonella*. These differential modes of depletion of commensals by CF pathogens are qualitatively summarized in Table 4 and shown by sample in Fig. 7B and Data Sets S1 and S2. We moreover noted that microbial communities cocolonized by S. aureus and P. aeruginosa typically had one species as the prime and the other one as only a minor member (Fig. 7B).

**FIG 8:**
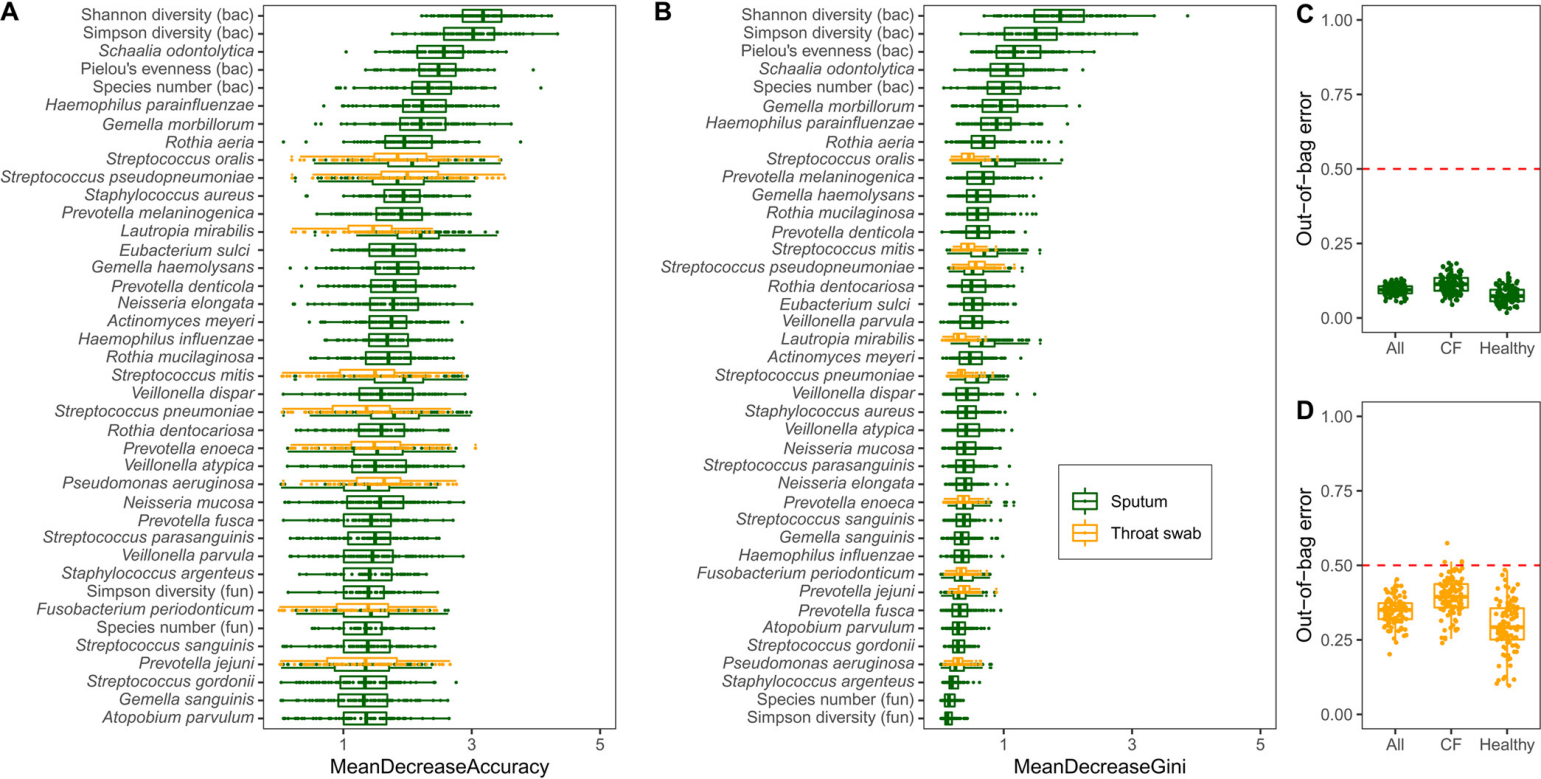
Random forest bootstrapping aggregation identified nonrandom key variables distinguishing healthy from PI CF cross-sectional sputum samples (green; *n* = 30) or throat swabs (orange; *n* = 15). (A) Representation of variable importance as defined by the mean decrease accuracy. (B) Representation of variable importance as defined by the mean decrease in Gini. (C and D) Outcome of the out-of-bag estimates of error for random forest classifications, which were repeated 100 times with different seeds set for the classification and Boruta feature selection. Two hundred ninety-four input variables were selected for the classification, including numerical ecological parameters of the bacterial and fungal community (Simpson diversity, Pielou’s evenness indices, Shannon diversity, and species number), subject’s metadata (gender and age), and bacterial and fungal metagenome data at the species level.

Next, we applied machine learning by the random forest method (62) to elucidate the key parameters among 294 microbial and host metadata that globally discern samples from CF and healthy donors (Fig. 8). The numerical ecological parameters of the bacterial community (Shannon diversity, Simpson diversity, Pielou’s evenness indices, and species number) were identified as the major classifiers of sputum specimens followed by a large number of bacterial species (Fig. 8). Conversely, as indicated by large out-of-bag errors, none of the host and microbial input variables reliably differentiated samples from PI and PS patients and oropharyngeal swabs from healthy and CF subjects from each other.

### Inflammation and microbiota in CF sputum.

Next, we sought associations between the patient’s lung function, inflammation and bacterial metagenome. Sputum specimens utilized for metagenome sequencing were examined for inflammatory mediators that are known to be diagnostic for the intensity of inflammation of CF lungs (63). Sputum levels were determined for the proinflammatory cytokines interleukins 1β, 6 and 8, the neutrophilic enzymes myeloperoxidase and elastase, matrix metalloproteinase 9, and metallopeptidase inhibitor 1, involved in the reorganization of the extracellular matrix (Data Set S3). According to principal-component analysis, the CF host’s lung function explained about 10% and the levels of the selected inflammation markers explained about 18% of the variance of the data sets within the groups of PI and PS CF patients (Tables 5 and 6). The remaining variance of the components was assigned to the abundance of the tested bacterial species.

**TABLE 5:** Principal-component analysis of inflammatory parameters, clinical data, and the top bacterial species in sputum of 28 PI CF patients^*a*^

Parameter	Contribution (%) of observation in dimension								
	F1	F2	F3	F4	F5	F6	F7	F8	F9
Streptococcus mitis	9.1	0.9	1.4	2.6	0.9	0.5	0.3	0.5	0.4
Streptococcus gordonii	8.0	0.0	0.0	0.1	0.1	1.8	1.0	4.8	0.1
Neisseria meningitidis	4.6	2.4	1.7	0.4	0.0	0.2	2.6	0.1	2.6
Haemophilus parainfluenzae	5.1	2.3	1.7	0.2	0.0	0.1	5.4	0.2	3.2
Rothia mucilaginosa	3.4	0.5	0.1	0.2	0.0	7.3	5.6	1.3	0.5
NE	4.0	1.8	2.7	3.0	1.7	4.2	1.1	0.4	0.9
Streptococcus oralis	7.0	0.1	1.8	3.5	0.0	1.1	1.1	0.4	0.1
Streptococcus pseudopneumoniae	8.3	1.0	1.3	3.4	1.1	1.3	0.1	0.5	0.1
Streptococcus pneumoniae	7.7	0.8	1.3	4.2	1.1	1.6	0.0	0.7	0.0
Staphylococcus epidermidis	2.8	0.2	0.5	5.6	7.4	0.0	0.7	1.1	0.1
Cutibacterium acnes	2.9	1.2	0.0	0.1	9.2	0.4	2.3	1.9	1.8
Streptococcus salivarius	2.8	5.8	0.0	6.2	2.5	3.8	1.8	0.1	0.1
Actinomyces oris	2.5	5.1	1.8	1.6	0.3	3.1	5.4	0.9	0.8
Actinomyces meyeri	2.2	4.5	2.2	2.0	0.1	4.0	6.4	0.3	0.7
Streptococcus parasanguinis	3.3	8.6	0.2	2.8	0.7	0.7	0.5	0.7	0.5
TIMP1	0.3	12.2	0.0	2.2	0.0	5.7	1.8	1.0	0.3
Streptococcus sanguinis	1.0	11.2	0.3	0.8	0.0	5.3	2.8	1.0	0.0
FEV1	0.7	9.3	0.1	0.5	0.3	4.7	5.5	2.8	3.2
FVC	0.6	7.5	0.6	4.3	0.2	1.3	3.1	0.5	2.1
Stenotrophomonas maltophilia	1.5	3.3	0.0	10.7	1.8	1.4	0.0	8.2	0.1
Age	0.6	7.6	0.6	8.1	0.0	0.4	0.1	1.5	1.1
Haemophilus influenzae	0.9	0.2	8.2	0.3	1.5	0.0	0.0	0.0	0.0
Streptococcus thermophilus	2.2	2.1	6.5	0.1	1.1	8.1	0.2	2.3	1.4
Rothia dentocariosa	1.2	0.6	10.4	3.9	0.6	5.4	6.9	0.6	0.8
MMP9	1.1	0.7	9.9	3.1	0.8	4.9	5.8	0.9	1.9
IL-1β	1.1	0.7	11.5	2.5	0.7	6.2	7.1	0.6	0.3
Streptococcus anginosus	0.0	0.4	8.1	0.1	0.5	7.1	8.5	0.1	0.7
MPO	1.1	0.6	3.8	0.1	0.0	3.0	5.4	12.3	0.2
Pseudomonas aeruginosa	1.2	0.6	9.6	0.2	0.4	2.8	0.2	7.0	0.1
Fusobacterium nucleatum	0.7	0.6	2.6	2.4	7.9	2.2	0.5	6.6	1.4
Veillonella parvula	0.1	0.8	3.2	0.0	19.9	0.1	0.0	0.4	7.9
Achromobacter xylosoxidans	1.2	0.7	0.1	3.4	7.0	4.2	0.1	0.4	1.0
Atopobium parvulum	1.7	2.6	1.8	4.8	7.3	1.7	0.6	3.7	1.9
IL-6	0.4	0.0	0.5	1.8	5.4	0.5	1.7	2.6	20.5
MEF25	0.6	1.9	0.0	0.1	2.9	0.8	0.6	10.2	2.6
Staphylococcus aureus	1.6	0.8	0.6	2.7	1.1	0.7	2.0	16.9	1.0
IL-8	0.3	0.0	0.0	0.1	0.9	0.6	4.4	1.6	31.6
Bifidobacterium longum	0.8	0.0	1.7	0.0	0.5	0.3	0.2	0.1	6.4
Prevotella melaninogenica	0.2	0.3	0.3	0.5	0.8	1.1	3.1	0.7	0.1
Variability (%)^*b*^	18.7	13.0	8.6	8.5	6.8	6.1	5.9	5.4	4.3
Cumulative (%)^*c*^	18.7	31.8	40.4	48.9	55.7	61.8	67.8	73.2	77.5

a The selected clinical parameters are age and the lung function parameters FEV1, FVC, and MEF25. The selected host defense molecules are IL-1β, IL-6, IL-8, neutrophilic elastase (NE), myeloperoxidase (MPO), metalloproteinase 9 (MMP9), and metallopeptidase inhibitor 1 (TIMP1).

b Explained variance by the respective principal component.

c Sequential sum of explained variance sorted in ascending order.

**TABLE 6:** Principal-component analysis of inflammatory parameters, clinical data, and the top bacterial species in sputum of 10 PS CF patients^*a*^

Parameter	Contribution (%) of observation in dimension				
	F1	F2	F3	F4	F5
Actinomyces meyeri	0.3	0.2	1.7	18.1	0.2
IL-8	2.6	2.2	0.3	8.2	0.2
Tannerella forsythia	2.5	1.4	0.4	9.2	1.7
Staphylococcus aureus	0.7	0.0	1.7	7.9	3.4
Bifidobacterium longum	1.7	0.0	0.0	5.1	0.0
Streptococcus anginosus	0.6	0.3	0.1	4.8	1.9
Achromobacter xylosoxidans	0.8	1.2	1.4	3.7	2.2
MPO	0.7	0.0	9.5	0.2	1.3
Fusobacterium nucleatum	2.5	1.1	3.1	4.2	0.1
Streptococcus pneumoniae	0.3	1.0	4.7	4.1	4.1
MMP9	1.8	0.8	3.4	0.1	11.2
TIMP1	0.2	2.9	4.0	0.6	12.2
Streptococcus gordonii	0.3	10.6	0.6	1.0	1.1
Pseudomonas aeruginosa	0.2	8.2	0.9	3.1	0.0
Haemophilus influenzae	0.6	8.0	0.1	3.0	0.6
Prevotella denticola	0.6	7.0	0.0	2.7	0.5
Streptococcus sanguinis	0.3	10.0	0.3	3.4	0.8
NE	1.3	7.1	3.3	1.0	0.3
Streptococcus thermophilus	5.8	0.3	4.4	0.1	0.4
Streptococcus salivarius	5.5	0.0	5.7	0.3	0.2
Streptococcus parasanguinis	5.5	0.0	5.7	0.3	0.2
Actinomyces oris	5.5	0.0	5.8	0.3	0.2
Atopobium parvulum	5.5	0.0	5.8	0.3	0.2
FVC	5.4	0.0	5.8	0.3	0.2
MEF25	4.8	0.3	6.9	0.0	0.0
Age	4.7	0.5	3.4	0.6	0.2
Streptococcus oralis	4.7	0.5	2.9	0.0	0.0
Rothia mucilaginosa	4.5	0.7	3.1	0.1	0.1
Prevotella melaninogenica	4.4	1.2	1.7	0.0	0.9
Veillonella parvula	4.4	1.3	0.0	0.0	10.3
Cutibacterium acnes	4.1	2.9	0.3	0.7	4.3
Streptococcus mitis	4.0	4.3	1.1	0.6	0.8
FEV1	5.0	0.9	1.6	4.6	0.1
IL-6	3.0	0.1	0.8	4.7	5.6
Rothia dentocariosa	1.2	0.7	0.0	0.9	4.6
Haemophilus parainfluenzae	1.0	1.9	0.1	0.0	19.5
IL-1β	2.9	0.5	1.6	0.0	0.0
Variability (%)^*b*^	28.3	17.8	14.2	10.3	9.2
Cumulative (%)^*c*^	28.3	46.1	60.2	70.5	79.7

a The selected clinical parameters are age and the lung function parameters FEV1, FVC, and MEF25. The selected host defense molecules are interleukins IL-1β, IL-6, and IL-8, neutrophilic elastase (NE), myeloperoxidase (MPO), metalloproteinase 9 (MMP9), and metallopeptidase inhibitor 1 (TIMP1).

b Explained variance by the respective principal component.

c Sequential sum of explained variance sorted in ascending order.

## DISCUSSION

CF airway ecology has been investigated by the 16S rRNA gene profile of bacterial taxa (5–8, 10–26, 61) and by WGS sequencing of the metagenome (3, 34–36, 38–48, 50). A common rationale for metagenome sequencing is the identification of the genetic content and the functional potential of the microbiota, particularly if most taxa of the habitat of interest are still unknown (33, 64). In the case of the human airway, however, completely sequenced reference genomes are already available for almost all bacterial taxa that reside in this habitat. The functional potential of the microbiome can thus be more reliably explored from annotated reference genomes than from metagenome sequence data sets, which, because of their insufficient coverage, will preclude genome assembling, at least from the rare species in the community (65). Instead, as described in this report, untargeted airway metagenome sequencing has value in the determination of the abundance of microbes (3) and the sensitive and specific identification of poorly cultivatable, rare, and closely related species (65, 66). The quantitative taxonomic profile of core and rare species can then be exploited for a comprehensive evaluation of the microbial community.

The published CF airway metagenome studies focused primarily on adolescents and adults with established lung disease characterized by the vicious circle of infection, inflammation, and airway remodeling (1, 67). Our metagenome data sets presented in this report and the recently published report on CF infants (50) cover all age groups. Since the disease centile distribution in our cohort matches that of the European registry (52), the results of this single-center study should be representative of the current CF population.

The microbial community was found to be rather loosely structured during the first year of life. During the second and third years, we identified stable microbial communities that were indistinguishable from those of a healthy infant (50). However, by the age of 4 years, the CF disease-specific signature of the lung metagenome started to emerge. By comparing the individual CF metagenomes of older patients (8 to 60 years) in this study with the average metagenome of healthy non-CF persons, it became evident that the spectrum of commensal bacteria in CF sputa was qualitatively and quantitatively different from that in sputa from healthy donors, even in the absence of any typical CF pathogen (Fig. 7A). Since sampling, sequencing, data processing, and evaluation of CF and non-CF specimens were performed at the same site, we assume that the methodological and technical bias is acceptable and that the divergent spectrum of commensals in the respiratory secretion of healthy and CF people reflects differential living conditions for the microbial community in CF and non-CF conducting airways.

The basic defect in CF, which from early on leads to mucus obstruction, anomalous pH of the airway surface liquid, a compromised innate, and an activated adaptive immunity (2), modifies the airway microbiome when people with CF are apparently healthy, have normal spirometry, and do not harbor any of the CF lead pathogens. Later, when the common CF pathogens take up residence in the lungs, we observed differential modes of depletion of the commensal microbiota in the presence of S. aureus, P. aeruginosa, S. maltophilia, or combinations thereof (Fig. 7). Variable CF habitats and divergent microbial lifestyles and microbe-microbe and host-microbe interactions should account for the differential evolution of the CF airway metagenome, which finally merges to uniform bacterial monocultures in end-stage lung disease. To the best of our knowledge, the varied depletion of commensals in the CF airway metagenome has yet not been reported. Since the loss or persistence of selected taxa could represent biomarkers of dysbiosis and its degree of severity, we encourage our peers to execute a replication study at another site. It will be of particular interest to learn whether combination therapy with effective CFTR modulators (68, 69) could prevent or slow the fragmentation of the CF airway metagenome in preschool children, when its composition is closest to that in healthy lungs (50, 65).

In summary, from early on, the CF airway metagenome evolves differently from that of the healthy non-CF population. Consistent with this conclusion, microbial ecology and selected bacterial taxa were the key classifiers that differentiated CF from non-CF sputa by the random forest algorithm (Fig. 8). Likewise, the individual profile of a patient’s sputum metagenome was more strongly shaped by the bacteria than by the CF host’s lung function and his local inflammatory response (Tables 5 and 6).

Besides these more general implications of this work, there are some specific findings worth mentioning, because they enrich or modify our understanding of CF airway microbiology. First, if the typical CF pathogens S. aureus and P. aeruginosa belonged to the trio of most abundant species (1, 8), only one of them was among the most abundant. Although culture-dependent diagnostics often identifies both taxa, the metagenome data told us that the dominant species is typically more abundant than the other by 1 order of magnitude or more. Coexistence of and interaction between S. aureus and P. aeruginosa are less prevalent than deduced from detection rates documented in registries. S. aureus and P. aeruginosa were present in nasal lavage samples but were far less abundant than in sputum, and hence, they may not play a central role in the nasal metagenome. The sinuses and nose are suspected to represent the gateway for the CF pathogens, from which they invade the lower airway (55, 70). This scenario has been substantiated by numerous independent studies. However, within the upper CF airway, corynebacteria and skin commensals are the key residents. With the exception of H. influenzae, the oral cavity was devoid of typical CF pathogens, consistent with our knowledge that the buccal epithelium does not express CFTR and thus is not affected in CF.

The lungs are the major site of chronic infections. The Gram-negative CF pathogens were more prevalent in the lung microbiomes of PI than in those of PS patients. Conversely, the constituents of a healthy lung flora, i.e., *Veillonella*, *Prevotella*, and Streptococcus species were more common in PS than in PI lungs. These findings confirm the registry data based on culture-dependent diagnostics showing that the amount of CFTR activity is the key determinant for the microbial colonization of CF lungs (1, 71); i.e., PI patients carry *CFTR* mutation genotypes which confer null or minimal CFTR activity, whereas PS patients harbor *CFTR* genotypes which confer some residual CFTR activity, about 10 to 20% of wild type (51, 70, 72).

Our metagenome study has some limitations. First, RNA viruses are not yet identified by DNA-based pipelines. Second, due to technical constraints of the protocols, microbes with easy-to-lyse and hard-to-lyse cell walls, such as *Tenericutes* and mycobacteria, were not recovered in representative amounts (3, 49). Third, metagenome data are presented from a single CF center. In principle, a multicenter study would have been more informative; however, generating high-quality data would have been logistically challenging. We performed sampling and processing according to in-house standard operating procedures (SOPs). Samples were shock-frozen to −80°C within less than 30 s, stored at −80°C, and processed using meticulous precautions (49, 50). We are hence confident that our data reflect the metagenomes of the airway compartments. Sputum reflects not only the lung microbiota but also to some extent the oral microbiota. Bronchoalveolar lavage principally bypasses the oropharynx (73), but according to our experience, the procedure is prone to microbial contamination by the equipment and the lavage fluid. Alternatively, saliva and sputum have been sampled in parallel to estimate the relative proportions of the lung and oral microbiome in a sputum sample (5). As in this work, we will continue to use induced sputum (58) collected according to a SOP of the U.S. CF Foundation (74). The sequential sampling after 3-min cycles of inhalation of hypertonic saline yields specimens that from the second portion onward are virtually free of oral contaminants.

In conclusion, each CF patient carries a personalized microbiome in his airway that after birth is initially unstable, but subsequently develops into a stable close-to-healthy network, which then gradually declines and becomes increasingly fragmented from early school age on. The spectrum of commensals is different in health and CF, and it remains to be seen whether the CF microbiomes will change their temporal characteristics with the implementation of lifelong CFTR modulation from early on.

## MATERIALS AND METHODS

### Cohorts.

Individuals with CF were recruited from the CF clinic at Hannover Medical School, Germany. All patients had been regularly seen at the CF clinic since the age of diagnosis. All patients were clinically stable at the day of sampling and had not experienced any pulmonary exacerbation (75) in the preceding 4 weeks. The diagnosis of CF had been made by the detection of two disease-causing mutations in the *CFTR* gene (51) and elevated chloride concentrations in the Gibson-Cooke pilocarpine iontophoresis sweat test (76) or a Sermet score in the CF range of nasal transepithelial potential difference measurements (77, 78) and/or chloride secretory responses in the CF range of intestinal current measurements (79). Exocrine pancreatic status was assessed by the fecal elastase 1 test (80). The exocrine pancreas-insufficient (PI) patients were either homozygous for the most common CF mutation, p.Phe508del (51), or compound heterozygous for p.Phe508del and a loss-of-function class 1 mutation (51). The exocrine pancreas-sufficient (PS) subjects were either compound heterozygous for a PI- and a PS-conferring *CFTR* mutation or homozygous for a PS mutation (51). Information about the patients’ lung function, anthropometry, comorbidities, and treatment was extracted from the patients’ electronic files.

The healthy cohort included 84 males and 73 females of European descent living in the Hannover region. The age of the control subjects varied from 10 to 59 years (median, 24 years; inner quartiles, 16 to 37 years). All subjects were free from pulmonary medical conditions, acute infection, and other known illness or chronic disease at the time of enrollment. Prior to the collection of induced sputum and oropharyngeal swabs, the participants were asked to fill out a questionnaire regarding their height, weight, smoking status, allergies, living conditions, physical activity, and nutrition (Table S2). The cohort consisted of 8 smokers and 149 nonsmokers. The median body mass index (BMI) was 22.3 kg/m^2^ (inner quartiles, 19.4 to 24.9 kg/m^2^; range, 14.7 to 35.9 kg/m^2^).

The study was approved by the ethics committee of the Medizinische Hochschule Hannover (no. 1510-2012; no. 9299_BO_K_2020) and by the ethics committee of the Jena University Hospital (no. 4323-02/15). Informed consent was obtained from all adult study participants and from legal guardians in the case of minors.

### Lung function.

Spirometry was performed and forced expiratory volume in the first second (FEV1), vital capacity (VCmax), and mid-expiratory flow at 25% of VCmax (MEF25) were determined according to American Thoracic Society (ATS)/European Respiratory Society standards (ERS) standards (81). Percent predicted results were based on equations of the global lung initiative (82).

### CF disease centiles.

Patients’ BMI and FEV1 values were ascertained on sample collection day and at all visits to the CF clinic at half-year intervals prior to and after taking the sample. Average values were mapped onto the age- and gender-corrected centile distribution of the 2013 edition of the CF European registry (52). Next, the FEV1 and BMI centiles were combined into a disease centile, which gives equal weight to lung function and anthropometry:

$$\text{disease centile}=\frac{\sqrt{{\left(\text{FEV1 centile}\right)}^{2}+{\left(\text{BMI centile}\right)}^{2}}}{\sqrt{2}}$$

The issue of whether the disease centile distribution of the study cohort recruited from the CF clinic Hannover matched that of the current European CF patient population was tested as follows. If a population of *n* CF patients follows the theoretical centile distribution, one can allocate *n* equidistant rank numbers to these individuals. If one determines all possible rank number differences in this cohort, the rank number difference of 1 will occur *n* − 1 times, but the maximal rank number difference (*n* − 1) will occur only once. In other words, the frequency of rank number difference will linearly decrease with increasing rank number difference. To compare the disease centile distribution of the *m* individuals of the study cohort with the theoretical centile distribution, the centiles from 1 to 100 were divided into *m* equal intervals, representing the *m* rank numbers. The centile of each individual was converted into a rank number, and for each individual, the rank number differences with all other members of the cohort were counted. The plot of the counts versus rank number difference was then compared with the expected linear decline from a rank number difference of 0 to a rank number difference of *m* (Fig. 1).

### Sampling from CF patients and healthy non-CF controls.

Samples were collected at regular visits of the CF outpatient clinic or prior to the first dose of a 14-day course of elective intravenous antipseudomonal chemotherapy. The upper airways were explored by nasal lavage (63): First, the nose was cleared of excessive mucus and crusts by forceful exsufflation/blowing into a handkerchief. Then, the seated or standing patient extended the neck approximately 30° from the horizontal while 10 mL of isotonic saline was instilled in a nasal side during elevation of the soft palate and holding of breath. After 10 s, the subject bent forward and gently rinsed the nasal lavage fluid into a sterile specimen cup. The fluid was decanted into a precooled tube, shock frozen, and stored at −80°C. The oral cavity was sampled with cotton swabs close to the oropharynx without inducing a cough. The cotton tip was immediately clipped with scissors into a precooled Eppendorf tube and shock frozen at −80°C. Induced sputum was collected by four 3-min cycles during which the participant breathed aerosolized 6% hypertonic saline according to SOP 530.00 of the Cystic Fibrosis Foundation Therapeutics (CFFT) Therapeutics Development Network (TDN) (74). Sputum expectorated after the second, third, and fourth cycles was collected, shock frozen within less than 30 s at −80°C, and then stored at −80°C.

### Control samples.

To control for the performance of sequencing, a mock community of equal amounts of genomic GC-rich P. aeruginosa, AT-rich Streptococcus salivarius, and GC≈AT Escherichia coli DNA was sequenced.

### Measurement of cytokines and chemokines (83).

The concentrations of the soluble inflammatory mediators interleukin 1β (IL-1β), IL-6, and IL-8, matrix metalloproteinase 9 (MMP-9), and tissue inhibitor of metalloproteinases 1 (TIMP-1) were quantified via bead-based multiplexed assay (xMAP; Luminex, Austin, TX, USA) by using Milliplex MAP kits (human high-sensitivity HS TCMAG-28K kit, human MMP panel 2 HMMP2MAG-55K kit, and human TIMP panel 1 HTMP1MAG-54K kit; all from Merck Millipore, Darmstadt, Germany). Neutrophil elastase (NE) levels were measured by enzyme-linked immunosorbent assay (DEH3311; Demeditec Diagnostics GmbH, Kiel, Germany). Assays were performed following the manufacturers’ instructions.

### Processing of samples.

**(i) Induced sputum.** Fresh or thawed samples were diluted 1:5 with ice-cold 97.5% phosphate-buffered saline–2.5% mercaptoethanol (vol/vol) and incubated on ice for 2 h with shaking. The suspension was centrifuged (15 min; 3,800 × *g*; 10°C), and the pellet was dried for 10 s, dissolved at 4°C in 10 mL double-distilled water, and then incubated on ice for 15 min on a rocker switch. This cycle of centrifugation, drying, and incubation in distilled water was repeated twice. The pellet was dissolved in 1 mL 0.1 RDD buffer (Qiagen, Hilden) and incubated in two 0.5-mL aliquots with 60 U DNase I for 90 min at 30°C under shaking (350 rpm). The solutions were combined, diluted with 40 mL DNase buffer and centrifuged (15 min; 3,800 × *g*; 10°C). The pellets were washed three times with 10 mL SE buffer (75 mM NaCl, 25 mM EDTA, pH 7.4) each by centrifugation, then dissolved in 0.5 mL SE buffer, and pelleted again (10 min; 12,000 × *g*; 10°C). Subsequently, genomic DNA was purified according to the “hard-to-lyse-bacteria” protocol with the NucleoSpin tissue kit (Macherey-Nagel, Düren, Germany). DNA was stored at 4°C in Tris-EDTA buffer. The yield of double-stranded DNA was determined with a Qubit 1.0 fluorimeter with the Qubit dsDNA BR assay kit (Q32850; Agilent Technologies, Santa Clara, CA). This protocol was found to be an acceptable compromise to obtain some nonstoichiometric amounts of hard-to-lyse mycobacteria and fungi without losing all easy-to-lyse mycoplasms.

**(ii) Swabs.** Cells were removed from the swab tips by 15 min incubation in SE buffer with intermittent vortex mixing. After centrifugation (15 min; 14,000 × *g*), the DNA was extracted from the dissolved pellet by the hard-to-lyse-bacteria protocol with the NucleoSpin tissue kit.

**(iii) Nasal lavage fluid.** Cells were collected by centrifugation (15 min; 14,000 × *g*). Then DNA was extracted from the dissolved pellet by the hard-to-lyse-bacteria protocol with the NucleoSpin tissue kit.

### Library preparation and DNA sequencing.

For all samples collected from people with CF and oropharyngeal swabs collected from healthy people, fragment libraries were prepared with a NEBNext Ultra II DNA library preparation kit for Illumina (E7645) and NEBNext unique dual index primer pairs (E64405), with a total of six to eight PCR cycles. The Illumina NextSeq 500/550 platform was used for sequencing (high-output kit v2.5; 75 cycles, single-end reads; no. 20024906; Illumina, San Diego, CA). For the induced sputa collected from healthy subjects, fragment libraries were prepared with the Nextera DNA Flex library preparation kit (no. 20018704; Illumina) with index adapter set B (no. 20027214) and subsequent PCR amplification. Sequencing was performed on the Illumina NovaSeq platform (NovaSeq 6000 S1 reagent kit v1.5; 300 cycles, paired-end reads; Illumina no. 20028317).

### Taxonomic classification of the airway microbial community.

The whole-metagenomic-sequencing alignment pipeline Wochenende (version 2.0.0) was applied to the taxonomic classification of microbial community members (84). For each sample, raspir (version 1.0.2) was run to extract microbial species with uniform read distributions toward the reference genome (66). EukDetect enabled an assessment of the community of fungi and moulds from our metagenome samples (59). Read counts were RPMM normalized, accounting for variations in both sequencing depth across samples (reads per million reads) and chromosome length (reads per million reference bases) (Data Sets S1 and S2) (84). Alternatively, bacterial load was quantified by the ratio of bacterial cells to human cells (3) or semiquantitative qPCR for selected cases.

### qPCR.

qPCR was performed to determine independent of metagenome copy number estimates, the amount of DNA of different bacterial species and human DNA in the metagenome samples. qPCR targets were selected for human DNA and six bacterial species (S. aureus, P. aeruginosa, H. influenzae, Prevotella melaninogenica, Rothia mucilaginosa, and Veillonella atypica), which had been indicated as being present in many metagenome samples by the full sequencing approach. Target loci and the respective primers were selected after literature screening, choosing targets that were described to have been used in qPCR approaches for distinctive species identification and quantification from mixed DNA samples (see Table S3 for a list of targets, primer sequences, and references). qPCRs using SYBR green for product quantification were performed with the QuantiFast SYBR green RT-PCR kit (Qiagen) on a 7500 fast real-time PCR system (Applied Biosciences). The corresponding software (Life Technologies 7500 software v2.3) was used for monitoring fluorescence curves, determination of cycle threshold (*C_T_*) values, calculation of Δ*C_T_* values from samples and negative controls, and melting curve analyses of the products. Experiments were performed in 96-well plates with a 20-μL reaction volume per well. All qPCRs were done with 10 ng DNA template, 40 amplification cycles, and 60°C as the annealing temperature (see Table S3 for the exact reaction conditions and corresponding PCR program). Each reaction was executed at least in duplicate on the same 96-well plate to allow *C_T_* value determination from more than one well. Controls containing distilled water instead of template DNA were included in each 96-well plate for all primer pairs used in the respective plate. After the qPCR, the products were inspected by gel electrophoresis using 4% agarose gels. Primer pairs were initially tested in positive- and negative-control reactions (with DNA of single species as templates) in order to optimize primer concentrations but also to sort out primer pairs that generated interfering amounts of nonspecific products.

Overall, 21 different metagenome DNA samples were used as templates in qPCRs. For all of them, reactions were done with primers targeting human DNA and primers for one to six bacterial species, depending on the preceding sequencing results for these samples. In addition, qPCRs were performed using artificial DNA mixes as templates, containing human DNA and DNA of two to four bacterial species in various proportions (refer to Table S3 for composition). Besides serving as additional controls, these reactions were also applied for assigning *C_T_* values to known DNA amounts or copy numbers, respectively, of a species in a qPCR template.

A detailed overview of all tested template/primer combinations is shown in Table S4.

### Statistics.

For comparing two independent groups and more than two groups, the nonparametric Mann–Whitney *U* test and the Kruskal-Wallis rank test were applied, respectively. For two groups, the effect size *r* was calculated, which is the Mann-Whitney *U* test statistics divided by the square-root of the sample size. For more than two groups, the epsilon-squared effect size and the corresponding confidence intervals (CI) were obtained. The Conover-Iman test with the Benjamini-Hochberg adjustment (85) was used for multiple comparisons between group levels. R statistical software was used for data analyses, including the rcompanion package for statistical testing (86).

Principal-component analysis was performed with XLSTAT BASE. MetaPhlAn2 (87, 88) was used for taxonomic classification of normalized sequence data and for the construction of heat maps of the most abundant species. Bray-Curtis dissimilarity indices were obtained for nonmetric multidimensional scaling (89) (without autotransform adjustment, *k *= 3, stress < 0.2). A permutation test (envfit [89], permutations = 1,000) was used to establish relationships between the nonmetric multidimensional ordination axes and metadata variables.

We applied the random forest algorithm (62) to rank the importance of variables for differentiating samples originating from CF patients and healthy non-CF subjects. To reduce the random forest regression model’s complexity (62, 90), a Boruta wrapper algorithm (91) was applied beforehand, extracting uncorrelated, nonrandom, and nonredundant features from our high-dimensional data set. Two hundred ninety-four input variables were selected for the classification, including numerical ecological parameters of the bacterial and fungal community (Shannon diversity, Simpson diversity, Pielou’s evenness indices, and species number), subject’s metadata (gender and age), and bacterial and fungal metagenome data at the species level. The Boruta algorithm generated shadow attributes by randomly shuffling the original input variables and iteratively comparing the random with the corresponding original variables, so that nonrandom features of importance for the prediction were distinguishable from random feature assignments (65, 91). Outcome of the out-of-bag (OOB) estimates of error for random forest classifications were repeated 100 times with different seeds set for the classification and Boruta feature selection. All data analysis was performed in R (86).

### Data availability.

The metagenome sequencing data sets have been deposited in the European Nucleotide Archive (ENA) (study accession no. PRJEB38221, PRJEB52317, and PRJEB52822) hosted by the European Bioinformatics Institute (EMBL-EBI) (www.ebi.ac.uk). The pipeline Wochenende is available at https://github.com/MHH-RCUG/nf_wochenende. The data integration tool Haybaler is at https://github.com/MHH-RCUG/haybaler. Our reference masking tool Blacklister is at https://github.com/colindaven/blacklister. The reference database is publicly available from https://drive.google.com/drive/folders/1q1btJCxtU15XXqfA-iCyNwgKgQq0SrG4?usp=sharing. The STORMS checklist for this article is available from Zenodo (10.5281/zenodo.7053226).
