# Supplementary material for: The Cystic Fibrosis Upper and Lower Airway Metagenome
Source: Microbiol Spectr. 2023 Mar 9;11(2):e03633-22. doi: 10.1128/spectrum.03633-22 (PMC10101124; doi:10.1128/spectrum.03633-22)

Supplement

Legends of supplementary material

Tables (Excel files)

Table S1. CF patients' characteristics at the day of sampling (*CFTR* genotype, CF related diabetes, any previous sinus surgery, inhaled antimicrobials).

Table S2. Metadata of the healthy control subjects at the day of sampling (gender; age; height; weight; BMI; susceptibility to airborne allergens; smoking status; place of residence).

Table S3. Information on qPCR reactions.

Table S4. Overview on qPCR assays.

Supplementary Data Sets (Excel files)

Data set S1. Taxonomic profile of bacteria identified in nasal lavage, oropharyngeal swab and induced sputum collected from exocrine pancreatic insufficient (PI) people with cystic fibrosis. The tables provide the total number of sequencing reads assigned to human, fungi, bacteria and DNA viruses and the number of reads aligned to individual bacterial species. The identifier of a sample is given (from left to right) by origin (M, CF clinic Hannover), pancreatic status (PI, PS), age group (A, 7-17 years; B, 18 -27 years; C, 28 - 50 years), gender (M, male; W, female), patient number; sample number; habitat (N, nasal lavage; T, oropharyngeal swab; S, induced sputum). FEV1, BMI and disease CF centiles were calculated from patients' height, weight and FEV1 at the day of sampling (t = 0) or from the average values of the assessments at all visits to the CF clinic during half-year periods prior and after the day of sampling (average).

Data set S2. Taxonomic profile of bacteria identified in nasal lavage, oropharyngeal swab and induced sputum collected from exocrine pancreatic sufficient (PS) people with cystic fibrosis. The tables provide the total number of sequencing reads assigned to human, fungi, bacteria and DNA viruses and the number of reads aligned to individual bacterial species. The identifier of a sample is given (from left to right) by origin (M, CF clinic Hannover), pancreatic status (PI, PS), age group (A, 7-17 years; B, 18 -27 years; C, 28 - 50 years), gender (M, male; W, female), patient number; sample number; habitat (N, nasal lavage; T, oropharyngeal swab; S, induced sputum). FEV1, BMI and disease CF centiles were calculated from patients' height, weight and FEV1 at the day of sampling (t = 0) or from the average values of the assessments at all visits to the CF clinic during half-year periods prior and after the day of sampling (average).

Data set S3. CF sputum levels of interleukins 1ß, 6 and 8, the neutrophilic enzymes myeloperoxidase and elastase and the matrix metalloproteinase 9 and the metallopeptidase inhibitor 1.

Figure S1. Venn diagram: Sample types collected per study subject. Supplementary data sets S1 and S2 provide the information about patient number, sample number and sample type (nasal lavage, oropharyngeal swab, induced sputum) separately for each sample.

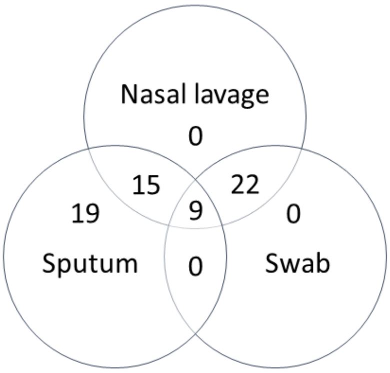

Supplement: Supplemental file 1 — Supplemental material. Download spectrum.03633-22-s0001.pdf, PDF file, 0.5 MB [file spectrum.03633-22-s0001.pdf]
